# The evolution and diversity of actin-dependent cell migration

**DOI:** 10.1091/mbc.E22-08-0358

**Published:** 2023-10-31

**Authors:** Lillian K. Fritz-Laylin, Margaret A. Titus

**Affiliations:** a Department of Biology, University of Massachusetts, Amherst, MA 01003; b Department of Genetics, Cell Biology and Development, University of Minnesota, Minneapolis, MN 55455; University of California, Berkeley

## Abstract

Many eukaryotic cells, including animal cells and unicellular amoebae, use dynamic-actin networks to crawl across solid surfaces. Recent discoveries of actin-dependent crawling in additional lineages have sparked interest in understanding how and when this type of motility evolved. Tracing the evolution of cell crawling requires understanding the molecular mechanisms underlying motility. Here we outline what is known about the diversity and evolution of the molecular mechanisms that drive cell motility, with a focus on actin-dependent crawling. Classic studies and recent work have revealed a surprising number of distinct mechanical modes of actin-dependent crawling used by different cell types and species to navigate different environments. The overlap in actin network regulators driving multiple types of actin-dependent crawling, along with cortical-actin networks that support the plasma membrane in these cells, suggest that actin motility and cortical actin networks might have a common evolutionary origin. The rapid development of additional evolutionarily diverse model systems, advanced imaging technologies, and CRISPR-based genetic tools, is opening the door to testing these and other new ideas about the evolution of actin-dependent cell crawling.

## THE MANY INDEPENDENT EVOLUTIONARY ORIGINS OF CELL MOTILITY

Archaeal, bacterial, as well as eukaryotic cells can swim through liquids and travel across solid surfaces to hunt prey, evade predation, and find mates. Cell biologists have been trying to understand how cells move as early as the 1600s, when van Leeuwenhoek observed cells moving through his samples. We now classify cell motility into four broad categories: 1) swimming, 2) gliding, 3) walking, and 4) crawling, each of which has evolved multiple times across the tree of life.

Although eukaryotes, bacteria, and archaea all swim using whip-like structures called “flagella”, these appendages evolved independently in each domain of life. Eukaryotic flagella (also called cilia) are complex, membrane-bound machines built around microtubules that are slid back and forth by dynein-motor proteins to power motility through bending (Mitchell, 2017). In contrast, bacterial flagella are assemblages of extracellular proteins called flagellins whose movement arises from the rotation of a transmembrane motor complex anchored to the cell body (Nakamura and Minamino, 2019). Archaeal flagella (also called archaella) are composed of completely different protein components, yet use a similar mechanism to bacterial flagella, representing a clear case of convergent evolution (Albers and Jarrell, 2015).

Like swimming motility, there are multiple independent evolutionary origins of gliding and walking motility. While swimming involves movement through a water column, gliding and walking motility each depend on interactions with solid surfaces: gliding motility involves stable contact with the surface and walking involves transient association by way of structures that attach and detach from the substrate. For example, bacterial gliding can be powered by motors anchored to the cell wall or by exuding slime that facilitates hydrodynamic-force production (Spormann, 1999). Eukaryotic cells also use a diverse array of gliding mechanisms, including attaching flagella to solid surfaces to propel themselves across surfaces using intraflagellar motors (Shih *et al.*, 2013). Other cells, like *Toxoplasma gondii*, lay down specific molecules onto environmental surfaces and then use them as handles to pull themselves along (Frénal *et al.*, 2017). Similarly, walking motility can be driven by independent mechanisms that clearly evolved separately, by actin-filled linear cell protrusions called filopodia as in *Choanoflagellates* and *Capsaspora* (Dayel *et al.*, 2011; Parra-Acero *et al.*, 2020), or by bundles of eukaryotic flagella as in *Euplotes* (Lueken *et al.*, 1996; Erra *et al.*, 2001; Larson *et al.*, 2022).

**FIGURE 1:**
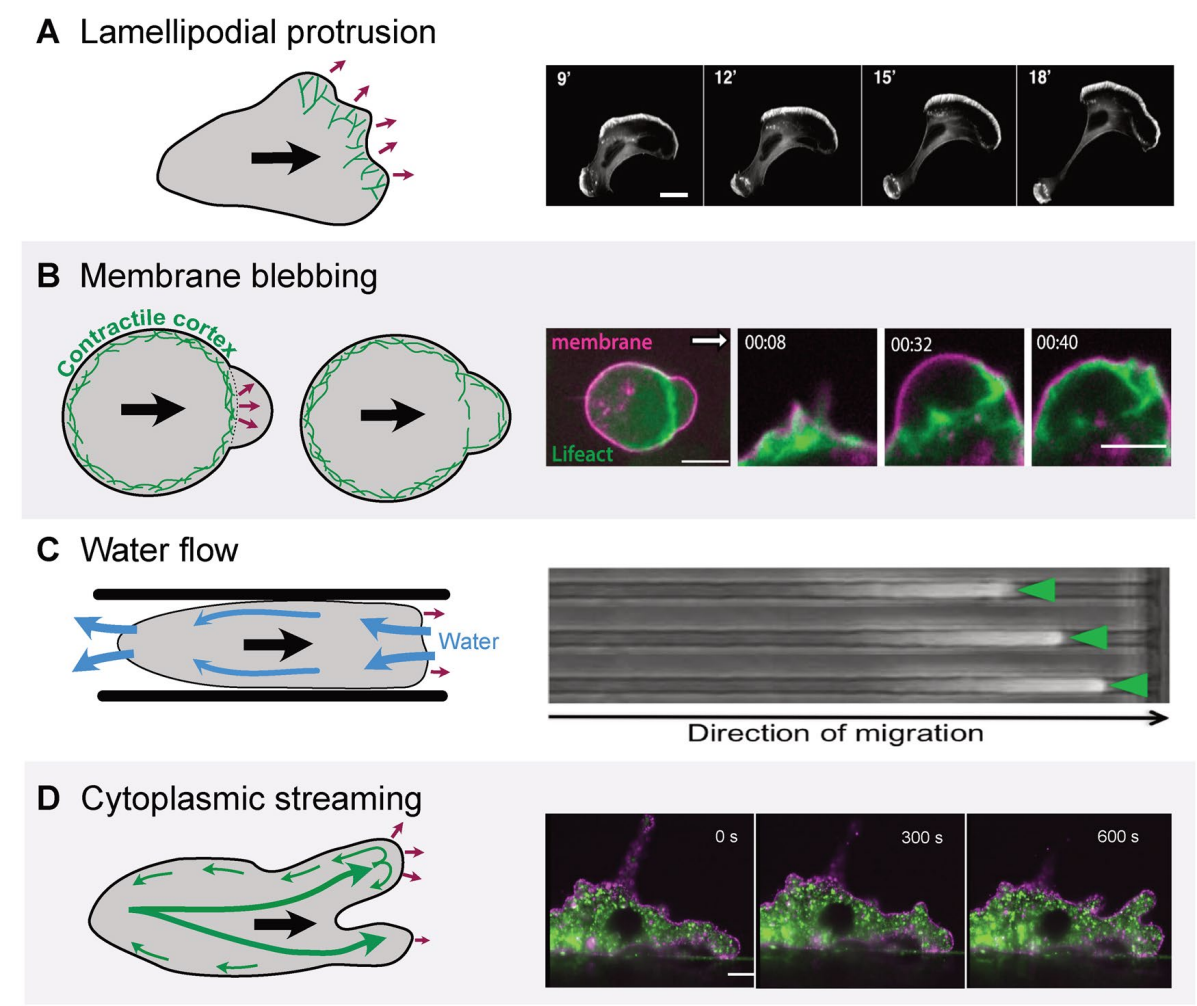
Diverse eukaryotic species crawl using multiple actin-dependent mechanisms. Eukaryotic cells can crawl using several distinct actin-dependent mechanisms. (A) Left: In lamellipodial protrusions, branched actin networks nucleated by the Arp2/3 complex push the leading edge forward. Right: Image of EGFP β-actin transfected B16 cells undergoing lamellipodial-based cell migration, scale bar = 10 µm (image adapted with permission from Ballestrem *et al.*, 1998). (B) Left: In blebbing motility, membrane delamination events that are powered by myosin II dependent contractility push the membrane of the leading edge forward, away from the underlying cortex. The actin cortex then regrows under the membrane. Right: select frames of a time lapse imaging sequence showing Zebrafish primordial germ cells undergoing blebbing motility, scale bar = 10 µm (taken from Olguin-Olguin *et al.*, 2021). (C) Left: Hydrodynamic flow can help push the membrane forward and enhance movement of cells undergoing actin-dependent cell migration by reducing loads experienced by actin networks at the leading edge. Right: Time lapse of a mouse S180 sarcoma cell exposed to hypotonic shock migrating in a 3 µm channel by hydrodynamic flow (image adapted with permission from Stroka *et al.*, 2014). (D) Left: Cells can also push their leading edges forward by cytoplasmic streaming that is mediated by myosin-II contractility at the rear. Right: Selected frames from a movie of an *Amoeba proteus* cell stained with DiI (membrane) and Mitotracker (mitochondria) extending pseudopodia via cytoplasmic streaming, scale bar = 30 µm (taken from Taniguchi *et al.*, 2023). Purple arrows indicate the directional movement of the membrane protrusion.

Cells can also crawl across solid surfaces using polymer network dynamics. While gliding and walking allow cells to maintain a constant cell shape, crawling motility requires cell-shape deformation by pushing out the cell membrane at the front while retracting at the rear. Although crawling is usually driven by actin, other polymers can be used, as evidenced by the crawling sperm of some worm species that rely on “major sperm protein” filaments (Roberts and Stewart, 1997). Crawling motility is essential to many aspects of animal biology, including embryonic development, immunity, and wound repair. Actin-dependent crawling motility of animal cells has been observed for well over a century (De Bruyn, 1947), and is now known to be driven by multiple, distinct-molecular mechanisms (Figure 1) that may originate from a single or multiple evolutionary sources.

**FIGURE 2:**
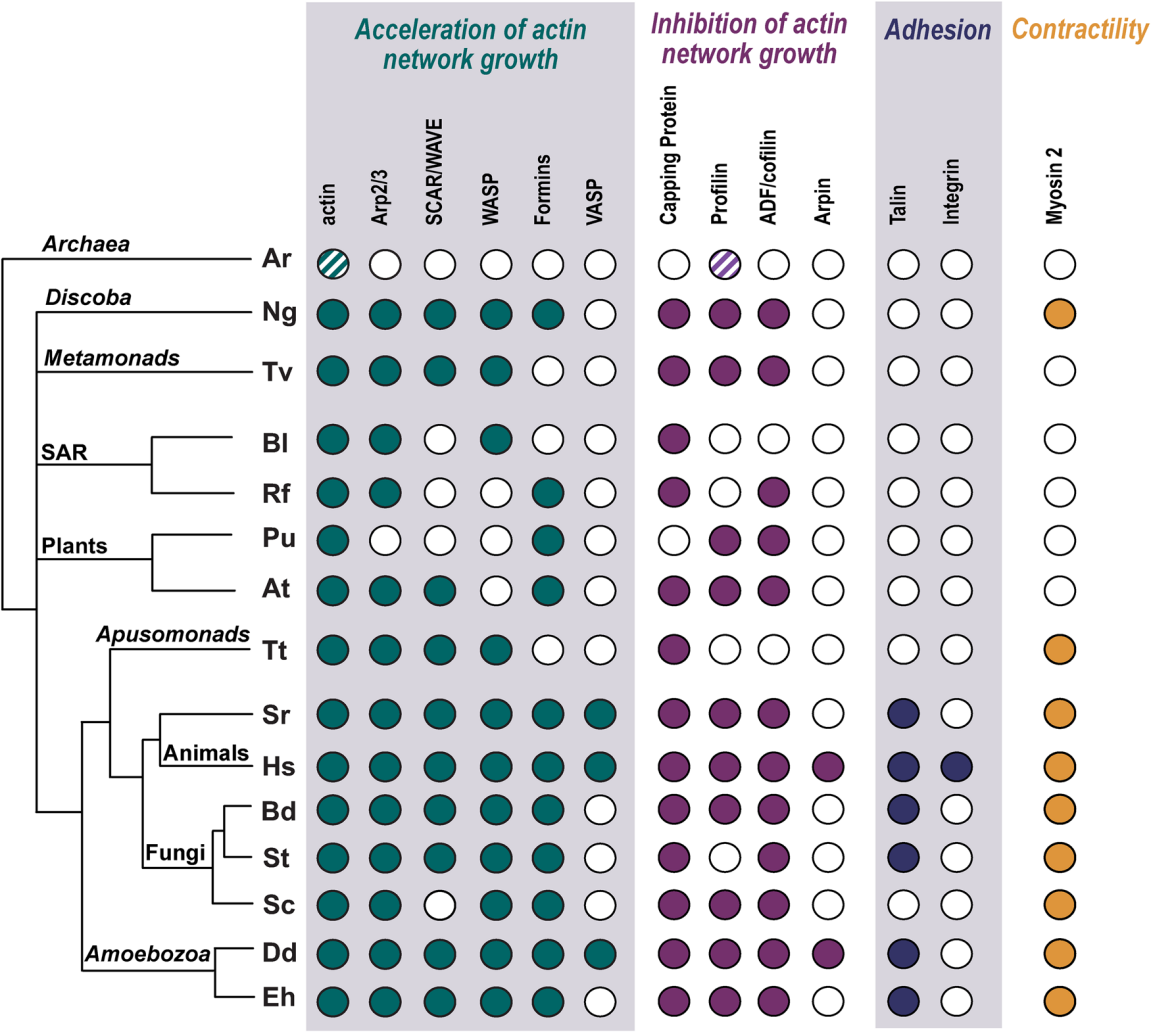
Actin regulators found in evolutionarily diverse eukaryotic cells that exhibit actin-dependent crawling motility. All species shown, except *Archaea* (Ar), *Arabidopsis thaliana* (At), *Reticulomyxa filosa* (Rf), and *Sacchromyces cerevisiae* (Sc) have been documented to undergo some form of actin-dependent cell migration. The presence of key regulators that accelerate or inhibit the growth of actin filaments, and proteins involved in adhesion and contractility in representative organisms is indicated based on available literature and/or presence of a homolog in EukProtdb (Richter *et al.*, 2022), empty circles indicate that no homolog has yet been identified. Striped circles indicate archaeal proteins with related structures and similar biochemical activities. Shown are - *Archaea* (Ar) including sequences from four phyla (Heimdall, Loki, Odin and Thor), *N. fowleri gruberi* (Ng), *T. vaginalis* (Tv), *Bigelowiella longifila* (Bl), *Reticulomyxa filosa* (Rf), *Porphyra umbilicalis* (Pu), *Arabidopsis thaliana* (At), *Thecamonas trahens* (Tt), *Salpingcoeca rosetta* (Sr), *Homo sapiens* (Hs), *E. histolytica* (Eh), *D. discoideum* (Dd), *Sacchromyces cerevisiae* (Sc), *Batrachochytrium dendrobatidis*, (Bd), *Sanchytrium tribonematis* (St). Information is taken from published papers and available genome sequencing data. Protein absence may be due to true biological absence, an overlooked gene due to incomplete genome sequencing and/or annotation, or protein sequence divergence. References for genome data: Bd, Sc, At, Dd, Hs - all in (Prostak *et al.*, 2021), Pu (Brawley *et al.*, 2017), Ng (Fritz-Laylin *et al.*, 2010), Tv (Kusdian *et al.*, 2013), Eh (Rath and Gourinath, 2020), Ar (Rodrigues-Oliveira *et al.*, 2023), Rf (Glöckner *et al.*, 2014), Bl, St, Tt, Sr - data from the EukProt database (Richter *et al.*, 2022). Documentation of crawling motility: Pu (Goodson *et al.*, 2021), Tv (Kusdian *et al.*, 2013), St (Galindo *et al.*, 2021), Bd (Fritz-Laylin *et al.*, 2017), Eh (Guillén, 1996), Bl (Ota *et al.*, 2007), Ng (Fulton, 1970), Tt (Cavalier-Smith and Chao, 2010), Sr (Brunet *et al.*, 2021), Hs (Grinnell, 1982), Dd (Yoshida and Soldati, 2006).

Actin-based cell crawling is not limited to animals (Figure 2). Indeed, studies of amoebae were some of the first to explore the molecular mechanisms underlying actin-dependent cell migration (e.g., Jennings, 1904 and many others reviewed in De Bruyn, 1947). Important early work on amoebae cytoskeletons, including *Amoeba proteus*, *Physarum polycephalum*, *Acanthamoeba castellanii*, and *Dictyostelium discoideum*, uncovered molecular mechanisms that drive crawling motility in human cells, amoebae, and diverse other species. This work laid the foundation for understanding the molecular regulation of actin polymerization and its role in cell migration (reviewed in Pollard, 2022). More recent work has revealed additional eukaryotic lineages that undergo actin-dependent cell migration. Although *Choanoflagellates* – the closest unicellular relatives of animals – have long been known to swim, recent work has shown that these cells also undergo actin-dependent cell migration when confined (Brunet *et al.*, 2021). Fungal species, too, have been shown to crawl rapidly using actin-filled protrusions (Fritz-Laylin *et al.*, 2017; Galindo *et al.*, 2021; Toret *et al.*, 2022), as well as cells in other distant eukaryotic lineages such as *Naegleria gruberi* from the *Discoban* lineage and *Trichomonas vaginalis* of the *Metamonads* (Fulton, 1970; Kusdian *et al.*, 2013; Velle and Fritz-Laylin, 2020). These discoveries have sparked interest in understanding how the motility of these species are related, in terms of their molecular mechanisms as well as their evolutionary origins.

## THE MOLECULAR MECHANISMS UNDERLYING ACTIN-DEPENDENT CELL CRAWLING

To trace the evolutionary history of actin-dependent crawling, we must understand the molecular mechanisms that drive this complex behavior. Cell crawling generally requires three activities: 1) protrusion, the pushing out of the front of the cell, 2) retraction, the gathering up of the rear of the cell, and 3) adhesion or some other form of interaction with solid surfaces that provides the friction needed to transform the first two activities into locomotion. Eukaryotic cells have evolved a number of biophysically distinct mechanisms that use actin-polymer networks in different ways to meet these requirements (Figure 1).

The most commonly studied mode of cell migration is powered by localized polymerization of actin filaments at the leading edge of cells (Figure 1A). This type of actin-based cell crawling was formally defined by a series of monographs from Michael Abercrombie in the early 1970s, which set the agenda for the cell-migration field to this day and make for excellent reading (see Abercrombie, 1997). We now know that activation of the actin-nucleation activities of the Arp2/3 complex by nucleation promoting factors such as the SCAR/WAVE complex results in formation of dense, stiff dendritic networks of actin filaments that are branched at a ~70° angle (Figure 1A). The ongoing addition of monomers to branched filaments abutting the plasma membrane pushes the membrane outwards. This results in the formation of a thin, single-leading edge called a lamellipodium, or a complex series of leading edges often called a pseudopod (these definitions vary in the literature; Fritz-Laylin *et al.*, 2018). The leading edge is integrated into actin networks that span the length of the cell and that typically incorporate contractile myosin II motors. The contraction of these actomyosin networks at the back of the cell, accompanied by the growth of the lamellipodial actin network at the front of the cell, drives retrograde flow of actin that balances forward protrusion by actin polymerization. Actin filaments pulled to the rear of the cell by myosin II are depolymerized, allowing for recycling of actin monomers that can be reincorporated into the actin networks at the leading edge. Posterior myosin II contraction also provides the forces for retracting the rear of the cell.

An alternative mode of protrusion used for crawling motility is via “blebs”– protrusions that are created by rapid separation of the plasma membrane from underlying cytoskeletal networks (Figure 1B; Hogue, 1919; Paluch and Raz, 2013). These “blistering” events are powered by intracellular pressure from contraction of myosin II in the actin cortex, a layer of actin found just below the plasma membrane. Rapidly growing blebs may allow cells to navigate more easily through confined, complex three-dimensional environments than actin-filled protrusions do (Zhu and Mogilner, 2016). Although blebs are ultimately stabilized by repolymerization of the actin cortex beneath the membrane of the bleb, cells can generate new blebs to continue moving. A wide range of cells employ blebs, including primordial germ cells (Blaser *et al.*, 2006), immune cells (Grinnell, 1982), cancer cells (Cunningham *et al.*, 1992; Friedl and Wolf, 2003), and Amoebozoa such as *Entamoeba histolytica* (Maugis *et al.*, 2010). Notably, cells can switch between branched-actin driven and bleb-based protrusion (sometimes called a mesenchymal-to-amoeboid switch), facilitating migration through a diverse range of extracellular environments. This plasticity is successfully exploited by metastatic-cancer cells that invade through dense tissues (Bergert *et al.*, 2012), such as in the brain or the lymphatic system, and by immune cells (Renkawitz *et al.*, 2009) as well as free-living amoebae (Yoshida and Soldati, 2006).

Locomotion requires cells to couple leading edge protrusion with surface interactions, often mediated by modulated adhesion to the substrate. Cells must balance the assembly and disassembly of such adhesions to first gain traction to generate movement and then break adhesions to make forward progress. In animal cells and *D. discoideum*, for example, adhesion receptors near the front of the cell make critical, initial attachments that are mechanically linked to the actin cytoskeleton via a highly conserved cytoskeletal linker called talin (Critchley, 2009; Klapholz and Brown, 2017). Talin binds to cytoplasmic tails of adhesion receptors via its N-terminal FERM domain and the actin cytoskeleton via a high affinity I/LWEQ region at its C-terminus. Its central helical region extends as traction forces on the receptors are applied, revealing binding sites for other proteins, such as vinculin, that reinforce the receptor/cytoskeletal linkage. While integrins are the major adhesion receptors in animals, integrin receptors are not found in most other lineages, e.g., Amoebozoa. This is likely because unicellular species do not crawl in a molecularly defined environment, and therefore require more generalized adhesion interactions than integrins that are specific for defined extracellular-matrix components such as fibronectin. However, it should be noted that adhesion receptors with some characteristics of integrins such as talin-binding NPxY motifs in their cytoplasmic tails, have been identified in *D. discoideum* (Cornillon *et al.*, 2006), suggesting that this basic mechanotransduction machinery may have arisen early in evolution. Talin has not been identified in other lineages, including that of *Naegleria*, suggesting that alternative molecules or mechanisms mechanically couple adhesion receptors to the actin cytoskeleton in these species. But given the small number of organisms studied outside of Amoebozoa and Opisthokonts (fungi and animals), it remains possible that talin-like molecules are present in other, yet unidentified, crawling cell types.

Despite the importance of molecular adhesions to the actin-based crawling of many animal cell types, they are not an absolute requirement for crawling motility. For example, generation of a mouse mutant with leukocytes lacking all integrins led to the surprising finding that these cells could still rapidly migrate through tissues despite having no known receptors to initiate specific adhesions (Lämmermann *et al.*, 2008). Detailed studies of these cells in vivo and in vitro revealed that movement occurred when cells were moving in a tight 3D network of extracellular matrix and not on 2D surfaces. This result spurred a rush of studies of cells crawling in nonadhesive environments that revealed that many kinds of cells can move in confined spaces of varied topologies using either branched-actin driven protrusion (Lämmermann *et al.*, 2008) or a combination of myosin II driven blebbing and rearward flow of cortical-actin networks which creates rearward shear forces that propel the cell body forward (Figure 1, A and B). These cells can thereby move in a manner akin to ‘chimneying’– a climbing maneuver where one is wedged in a tight space between two rocks and moves by pushing downwards against the walls with hands and feet to propel oneself upwards. Likewise, nonadhesive cells squeezed into a tight space can generate outward pressure and friction against the sides using the force exerted by anterior protrusions to drive the cell forward. This mode of motility has been recapitulated in “artificial cells”– lipid droplets encapsulating lipid-bound actin networks (Sakamoto *et al.*, 2022). Increasing the contact area between the droplet and the surface increases friction and thereby the speed of the droplet. Conversely, reducing the contact area slows the droplet, as does inhibiting myosin II activity, which reduces rearward-actin flow. A key feature of this system is its independence from any specific adhesion receptors, meaning that cells that use this basic physical mechanism could move in environments of varied surface composition or topology using only a dynamic-actin cytoskeleton.

Nonadhesive cells can also move in confined environments via water permeation and osmotic swelling (Figure 1C). Differential localization of specific ion exchangers, ion channels, and aquaporins at the front (e.g., Na/H exchanger and AQP5) and rear (e.g., volume regulated Cl^–^ channel and AQP4) moves water from the front to the rear of the cell (Stroka *et al.*, 2014; Li *et al.*, 2020; Smith and Stroka, 2023). This biased flow of water is accompanied by an increase in the volume of the front of the cell and a shrinking of the volume at the rear of the cell, leading to cell migration (Stroka *et al.*, 2014; Zhang *et al.*, 2022). A similar mechanism is also seen in unconfined cells; neutrophils stimulated by chemoattractant swell and the movement of water through the cell promotes rapid migration in collaboration with actin polymerization at the leading edge (Nagy *et al.*, 2023), hinting that water flow may assist actin-based cell migration in general.

Cell crawling can also be powered by cytoplasmic streaming (Figure 1D). Early observations of crawling amoebae included descriptions of cytoplasmic flows that move toward the front of the cell, then split and travel backwards (De Bruyn, 1947). Later work showed that contractile forces at the rear of the cell, possibly generated by myosin II, push cellular contents forward and differences in cytoplasmic viscosity at the side and front of the cell correlate with faster or slower rates of streaming, respectively. The faster streaming at the front, in a region of low viscosity, pushes the cell membrane forward. In the case of *P. polycephalum* amoebae, coordination between traction stresses and cytoplasmic flow results in protrusion of the front of the cell and forward movement (Lewis *et al.*, 2015).

Taken together, the different mechanisms of crawling movement outlined above highlight the versatile actin-dependent mechanisms that have evolved in eukaryotic cells. These varied modalities are undoubtedly suited for the environments cells find themselves in, and the ability to switch between modalities endows cells with the capacity to readily navigate changing landscapes and environmental conditions.

## LOOKING BACK: WHERE DID ACTIN-DEPENDENT CELL CRAWLING COME FROM?

How and when crawling motility evolved and diversified across the eukaryotic tree remains largely mysterious. This uncertainty is in contrast to ciliary motility that clearly evolved after the eukaryotic lineage diverged from the archaeal lineage but before the last common eukaryotic ancestor. This evolutionary history has been traced by phylogenetic analyses of structural and regulatory proteins that can serve as a useful proxy for the evolution of the cilium itself because they are used only for cilia (Mitchell, 2017). To use this approach to determine when actin-based cell migration evolved, therefore, we must first understand the evolutionary history and phylogenetic distribution of the proteins that drive cell crawling (Figure 2). Although actin-dependent cell motility can be driven by several distinct mechanisms (Figure 1), all of these rely on membrane-associated Arp2/3 complex-derived branched actin networks that provide pushing forces that respond to load (Bieling *et al.*, 2016; Mueller *et al.*, 2017; Li *et al.*, 2022), and/or unbranched, formin-derived filaments that can cooperate with myosin motors to provide contractile forces (Reymann *et al.*, 2012). The actin regulators that control these networks are shared with a wide variety of other essential functions, including endocytosis, cytokinesis, and the actin cortex that supports the plasma membrane. Because there are no known evolutionarily conserved proteins used only for actin-based cell migration, it is challenging to study the evolution of this form of cell motility using a phylogenetic analysis of individual actin-cytoskeletal proteins (Fritz-Laylin *et al.*, 2017).

The structural and regulatory overlap between actin networks used for cell motility and those used for the actin cortex could offer clues about the evolution of actin-dependent crawling. Cells that undergo actin-dependent cell migration use their actin cortex to support the plasma membrane from within and to deform the plasma membrane during crawling motility. The actin regulators that drive actin-based cell migration also build and maintain the actin cortex, which is intimately connected to the networks used for motility; these cortical-actin networks are thought to form a backstop for Arp2/3 complex-dependent protrusions such as lamellipodia and pseudopodia. They also initiate the hydrostatic pressure required for bleb formation through myosin II-mediated contraction. Moreover, the actin cortex and the actin networks used for motility are both closely associated with the plasma membrane. This raises the possibility that cell migration is an emergent property of dozens of actin-network activities necessary for assembly and regulation of an actin cortex. Under this model, it appears simple for cells to redeploy membrane-supporting actin networks for cell migration as they would already have the necessary machinery built into the cell cortex.

One line of evidence for the idea that cortical-actin networks are the prima mater for cell migration can be found in lineages that have evolved cell walls, particularly the fungal kingdom. Multicellular fungi and yeast, which grow inside chitin-based cell walls, have a minimal actin cortex that is restricted to distinct actin patches that drive endocytosis. Because they evolved from ancestors that had life stages that lack a cell wall, and used thick, actin-rich cortices to crawl using actin-dependent cell migration (Prostak *et al.*, 2021), it appears that the loss of cell migration may have correlated with loss of an actin cortex. Plant cells are also encased in stiff cell walls and lack a thick actin cortex and actin-dependent cell migration, consistent with the need for the evolution and retention of an actin cortex underlying a deformable membrane for the ability of a cell to migrate.

## MOVING FORWARD: NONANIMAL LINEAGES HOLD THE KEYS TO UNDERSTANDING THE EVOLUTION OF CELL MOTILITY

We currently only understand the molecular mechanisms of actin-dependent cell migration in species from a handful of lineages, most notably animals and amoebozoans. Developing and testing new hypotheses about the evolution of actin-dependent crawling motility requires an understanding of the diverse molecular mechanisms driving actin-dependent cell crawling in a much wider variety of eukaryotic lineages. These neglected lineages include species with intriguing actin-based cell migration mechanisms that indicate that we are only just touching the surface of the diversity of mechanisms that power eukaryotic cell motility.

A likely source of novel mechanisms of crawling is the actin-based cell migration of organisms lacking myosin II. Although myosin II is central to the migration of animal and amoebozoan cells (where it also plays important roles in cytokinesis), most major eukaryotic lineages have no identifiable myosin II, yet include species that undergo actin-dependent cell migration (Figure 2). The metamonad *T. vaginalis*, for example, rapidly transitions from a swimming flagellate to a crawling amoeboid cell upon contact with an epithelial cell layer. While it seems likely that *T. vaginalis* uses actin-filled protrusions to extend leading edges (Kusdian *et al.*, 2013) how the rear ends of these cells manage to keep up with the front without myosin II is a mystery. Red algae also undergo an actin-based cell migration without myosin II *or* the Arp2/3 complex (Goodson *et al.*, 2021), raising additional questions about the mechanisms used at the front as well as at the rear in these species, and whether the rules learned from animals and their relatives are universal or phylogenetically limited.

Looking more broadly at cell migration may also reveal some actin-independent surprises. An intriguing example is provided by the sperm of *Ascaris* and *Caenorhabditis elegans* worms that lack actin and microtubules. These cells exhibit remarkable amoeboid migration that is powered by major sperm protein (MSP), a small protein that polymerizes into filaments without polarity in a pH-dependent manner (Roberts and Stewart, 1997). Higher pH at the front of the sperm favors MSP filament polymerization and bundling that pushes the leading edge forward while lower pH at the rear causes depolymerization, resulting in the MSP filament network treadmilling similar to what is seen for the actin network in many other cell types. MSP-dependent cell migration holds an important lesson: even cells that encode a full suite of actin regulators in the genome can assemble lamellar protrusions using distinct mechanisms that result from convergent evolution.

There is also growing interest in understanding the deep roots of actin-based cell migration, focusing on the origin and diversification of actin itself. Because actin and actin-related proteins (Arps) evolved before the last common eukaryotic ancestor, we must turn to species outside of eukaryotic lineages to look for the roots of these key protein families. Insights have emerged from studies of archaeal proteins with structural similarity to eukaryotic actin and Arps. These proteins assemble into helical filaments strikingly similar in structure to metazoan actins (Izoré *et al.*, 2016), some of which have been observed forming filaments in cellular extensions (Rodrigues-Oliveira *et al.*, 2023). Archaea also harbor other hypothesized actin-like regulators, such as a profilin-like protein (Akıl and Robinson, 2018) and a gelsolin-like protein with multiple activities including actin-monomer sequestration, bundling, and capping (Akıl *et al.*, 2020) hinting that some actin regulators and perhaps even a form of actin-dependent motility may have evolved before the divergence of eukaryotes from archaea.

Even with these recent discoveries, the major roadblock to understanding the evolution of actin-based cell crawling remains the small number of organisms studied. The migration of only a few species has been characterized in any molecular detail, and this work is highly skewed towards metazoans and related amoebozoans, leaving other lineages largely unexplored. For example, the SAR lineage is thought to be the most genetically diverse eukaryotic group, which includes kelp and various other algae, ciliates, dinoflagellates, and human pathogens, many of which exhibit diverse and interesting modes of cell motility. However, most SAR species are known by their DNA sequence alone and have never even been seen by human eyes. Moreover, SAR is only one of several poorly characterized groups that likely harbor surprising, new modes of crawling waiting to be discovered.

Despite the clear need for new model systems, few groups are taking on the challenge. Developing new model systems to study cell migration requires serious investment, including isolating species from different environments and developing culturing methods to grow and handle them. Once in culture, we can use an ever-growing variety of imaging modalities (e.g., live-cell imaging, expansion microscopy, and cryoelectron tomography) to identify the cytoskeletal assemblies and molecular mechanisms used by these diverse species to crawl. Such studies will benefit from an open-minded approach that moves beyond plain glass coverslips and allows cells to explore more complex environments that mimic their natural habitats. As studies of worm sperm have taught us, careful description of cellular protrusions and surface interactions must be coupled with molecular analysis of the mechanisms that drive migration. In addition to adapting transformation and CRISPR technologies to new species, there is a clear need for new cytoskeletal inhibitors that can work in nonanimal cell types. Many unicellular organisms have efflux pumps that expel toxins in natural environments as well as commonly used cytoskeletal inhibitors, rendering them ineffective for probing cytoskeletal functions. Other inhibitors are effective against phylogenetically limited targets. Development of the next generation of inhibitors that target cytoskeletal proteins in diverse lineages could also prove to be useful for medicine and combating agricultural pests. We look forward to these and other ongoing efforts to rapidly develop new model systems and what they will tell us about the diversity and evolution of actin-dependent cell migration.

## Abbreviations used:

AQP: aquaporin
Arp2/3: actin-related protein 2/3
eGFP: enhanced green fluorescent protein
I/LWEQ: isoleucine/leucine, tryptophan, glutamic acid, glutamine
MSP: major sperm protein
NPxY: asparagine, proline, any amino acid, tyrosine
SAR: Stramenopiles, Alveolates, Rhizaria
WAVE: Wiskott-Aldrich syndrome protein-family verprolin homologous FERM, band 4.1, ezrin, radixin, moesin
